# Optimal Cerebral Protection Confirmed by Transcranial Doppler During Transcarotid Artery Revascularization

**DOI:** 10.14797/mdcvj.1465

**Published:** 2024-12-26

**Authors:** Reka Vernes, Adam Bardoczi, Alan B. Lumsden, Zsolt F. Garami

**Affiliations:** 1 Houston Methodist Hospital, Houston, Texas, US; 2 Vascular Ultrasound Laboratory, Houston Methodist DeBakey Heart & Vascular Center, Houston, Texas, US

**Keywords:** transcarotid artery revascularization (TCAR), carotid revascularization, transcranial Doppler, cerebral emboli, stroke

## Abstract

Transcarotid artery revascularization (TCAR) is a novel method to treat severe stenosis of the carotid artery with minimal embolization. During TCAR, flow reversal system redirects blood from the internal, external, and common carotid arteries into the femoral vein through a filter system to prevent debris and microparticles from entering the cerebral circulation. Transcranial Doppler (TCD) monitoring allows real-time detection of blood flow in the cerebral arteries during the operation and informs the surgeon of flow changes or possible emboli. With this information, the steps and maneuvers during the procedure and the function of the flow reversal system can be further improved to avoid stroke or other neurological complications. In this case study, we present a TCAR procedure with TCD monitoring in an asymptomatic male patient exhibiting severe left-sided internal carotid artery stenosis. Optimal cerebral protection was achieved due to the neuroprotective flow reversal system of TCAR.

## Introduction

The 2022 guidelines from the Society for Vascular Surgery recommend surgical management alongside state-of-the-art medical treatment for patients with carotid stenosis exceeding 70%, even if they are asymptomatic, to reduce the long-term risk of stroke.^1^ The perioperative stroke rates during or after carotid endarterectomy (CEA) and transcarotid artery revascularization (TCAR) are comparable and significantly lower than those for transfemoral carotid artery stenting (TF-CAS).^2,3,4,5^ However, there are important factors to consider when individualizing therapy for certain patients. For example, one study has shown that undergoing TCAR shortly after a stroke significantly increases perioperative stroke and mortality rates compared to CEA.^6^

Although CEA remains the predominant procedure for carotid stenosis, TCAR is gaining traction in terms of both utilization and patient access. Hospitals that have incorporated TCAR into their surgical options have seen an overall reduction in perioperative major adverse cardiovascular event (MACE) rates.^7^ Perioperative stroke caused by dislodged emboli is a feared complication of all carotid surgeries. In CEA, this risk is mitigated using techniques such as back-bleeding and sequential unclamping (external carotid artery [ECA]→common carotid artery [CCA]→internal carotid artery [ICA]), whereas TCAR implements blood flow reversal and a 200-micron filter.^8^

### TCAR Procedure

During TCAR, the CCA is directly accessed via a small vertical or horizontal surgical incision, based on aesthetics. Reverse blood flow is established using a proprietary system developed by Silk Road Medical (Sunnyvale, CA). The blood flow is redirected from the ICA and ECA into the femoral vein through a filter system to prevent debris and microparticles from entering the cerebral circulation.^9^ The Safety and Efficacy Study for Reverse Flow Used During Carotid Artery Stenting Procedure (ROADSTER) multicenter trial has demonstrated that the perioperative and mid-term stroke rates after TCAR are comparable to those of CEA, leading to its broader utility.^5^

### TCAR Requirements

Anatomical criteria include the following:

- There must be at least 5 cm between the CCA access site and stenotic lesion.
- The CCA must be at least 6 mm in diameter and free of significant disease.
- The ICA must be at least 4 mm in diameter.

These criteria must be verified with preoperative imaging. Silk Road Medical also provides a preoperative ultrasound worksheet that displays all the important aspects that the sonographer must evaluate. Once complete, an objective decision can be made regarding the patient’s eligibility for TCAR. The company also provides support during the process if needed. Contraindications and limitations of the procedure are previous neck irradiation, neck immobility, kyphosis, or severe obesity.

### TCAR Timeout

After the arterial and venous sheaths are placed and connected, the surgeon clamps the CCA and turns the reverse flow to high. Before this essential step, the operating team performs a TCAR timeout. During this time, the surgeons and anesthesiologists review and reiterate that, going forward, systolic blood pressure remains between 140 and 160 mm Hg, the heart rate remains at or above 70 beats/min, and the activated clotting time remains at or above 250 sec.

### TCD Monitoring

Transcranial Doppler (TCD) is a low-cost noninvasive ultrasound modality used to provide real-time emboli and flow detection in the main intracranial arteries. Preoperative TCD detects the depth and direction of blood flow in the middle cerebral arteries (MCAs).^11^ This information is used to evaluate patients who may benefit from carotid interventions. Tracking the number of embolic signals, also known as high-intensity transient signals (HITS), over a certain time period (eg, 30 or 60 min) may predict the risk of a transient ischemic attack or stroke, which may direct the treatment plan for a patient with carotid disease toward surgical resolution.^10^ Additionally, TCD adds a new dimension to the static images obtained with preoperative computed tomography angiography (CTA) and magnetic resonance angiography (MRA) by adding dynamic flow metrics and pulse qualities. During TCAR, TCD provides useful information regarding the flow dynamics and severity of the proximal stenosis. Evaluation of the flow dynamics in the vessels of the circle of Willis, as well as the state of collateral circulation, may predict blood flow alterations in response to the CCA clamping by the surgeon. The real-time flow assessment and emboli count also mitigate the risk of procedure-related stroke by providing continuous feedback to the surgical team, thereby enabling them to quickly assess the need for any adjustments.^12,13,14^ Postoperative magnetic resonance imaging (MRI) has shown that microemboli during carotid procedures are linked to the development of new ischemic foci, along with neurological symptoms.^15^

At the beginning of TCAR, TCD is used to assess the baseline blood flow in the MCAs. During the operation, ongoing communication between the TCD team and the surgeons regarding the blood flow is critical, especially after clamping the CCA. Communication between the TCD operator and the anesthesia team is also essential to maintain sufficient mean arterial pressure to ensure proper cerebral flow. TCD also detects hyperemia, which can be managed by adjusting the mean arterial pressure. Because of these qualities, TCD is sometimes called the brain’s “blood pressure cuff.”

Here, we present a case in which TCAR is seamlessly performed with optimal cerebral protection from emboli on an 83-year-old male patient. View this case report in an intraoperative recording of the surgery, fluoroscopy, and transcranial Doppler (TCD) monitoring (Video 1), also available in the DeBakey CV Education video library on YouTube.

**Video 1:** Intraoperative recording of the surgery, fluoroscopy, and transcranial Doppler monitoring; see also at https://www.youtube.com/watch?v=cRSKdlaTNSQ&t=93s.

## Case Report

In 2021, an 83-year-old male presented to the emergency department twice with transient partial vision loss in his right eye, and his symptoms spontaneously resolved both times. His medical history was significant for hypertension, peripheral artery disease, stable abdominal aortic aneurism (4 × 5 cm), chronic pancreatitis, prostate and bladder cancer, glaucoma, and obstructive sleep apnea treated with continuous positive airway pressure. His surgical history was significant for carotid endarterectomy on the right side in 2021, right inguinal hernia repair, and recent prostate surgery. He was a current smoker with a 64-pack-year history.

In 2021, an assessment of the patient revealed severe carotid artery stenosis on both sides, with greater severity on the right. Carotid duplex ultrasound measured a peak systolic velocity of 398 cm/sec and an end diastolic velocity of 171 cm/sec, with a ratio of 4.93 and flow volume of 422 cc/min in the right ICA, indicating severe stenosis (80-99%).

**Figure 1:**
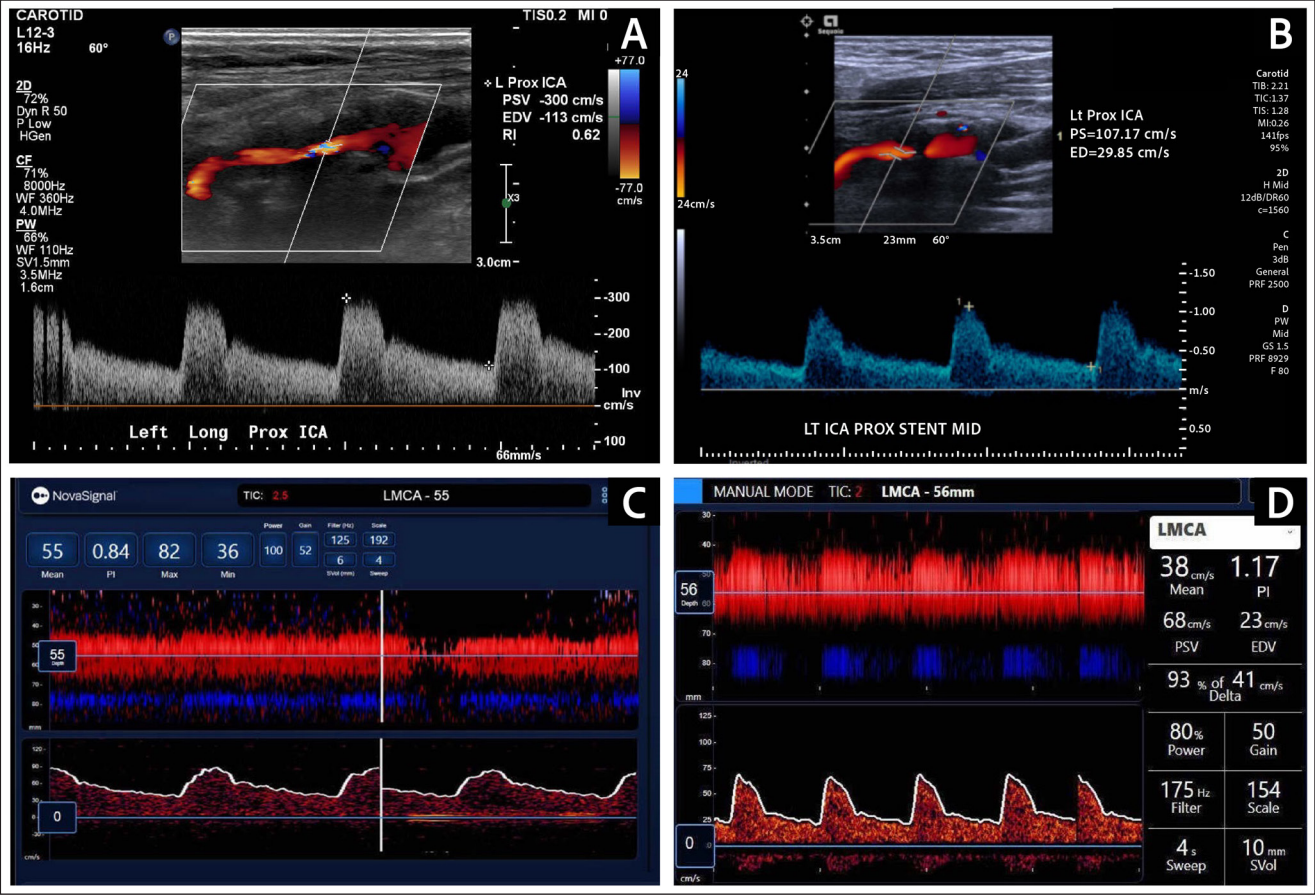
**(A)** Preoperative carotid Duplex ultrasound revealed severe stenosis in the proximal ICA (PSV of 300 cm/sec, EDV of 113 cm/sec). **(B)** Postoperative carotid Duplex ultrasound confirmed good stent patency, normal flow velocities (PSV of 107.17 cm/sec, EDV of 29.85 cm/sec) and improved pulse waveforms. **(C)** Preoperative TCD showed a blunted left MCA pulse waveform, confirming a proximal stenosis. **(D)** TCD after stenting revealed substantial improvement in the systolic upstroke in the pulse waveform of the left MCA. ICA: internal carotid artery; PSV: peak systolic velocity; EDV: end diastolic velocity; TCD: transcranial Doppler; MCA: middle cerebral artery

Due to the severity of the lesion in the right ICA, CEA was performed in 2021. Because stenosis was also detected in the left ICA, his follow-up plan included a left CEA, but it was delayed due to the COVID-19 pandemic. In 2023, carotid duplex ultrasound reconfirmed severe stenosis in the left ICA. It measured a peak systolic velocity of 300 cm/sec and an end diastolic velocity of 113 cm/sec, with a ratio of 3.93 and flow volume of 172 cc/min, indicating severe stenosis (70-99%). No stenosis was detected in the right ICA (Figure 1A).

The preoperative TCD in 2023 revealed increased velocity in the right anterior cerebral artery and a blunted left MCA pulse wave, confirming left ICA disease (Figure 1C). The flow in the right anterior cerebral artery was higher than that in the right MCA, suggesting a right-to-left collateral circulation. Antegrade flow was detected in the vertebrobasilar system and in the ophthalmic arteries.

**Figure 2:**
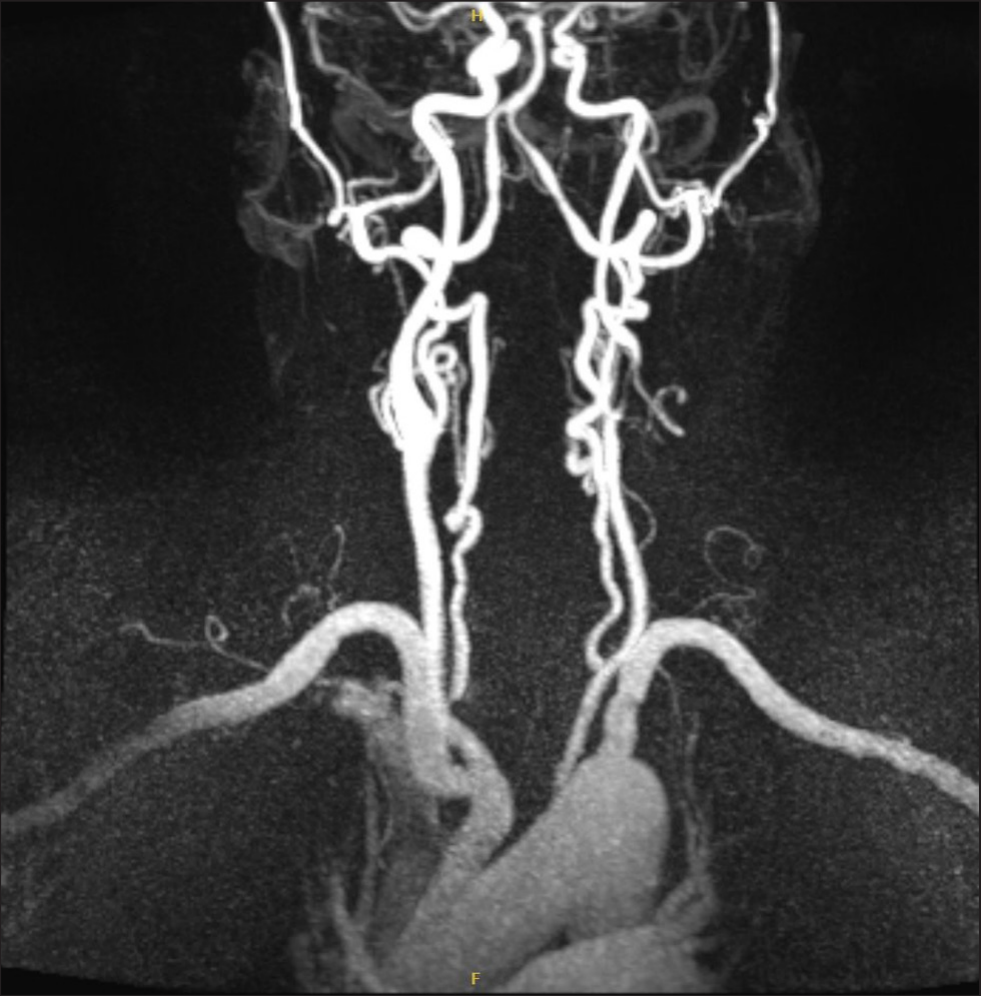
Magnetic resonance angiogram revealed significant left internal carotid artery stenosis (70-80%).

MRA of the neck revealed severe narrowing of the left cervical ICA, estimated at 70% to 80% stenosis by North American Symptomatic Carotid Endarterectomy Trial (NASCET) criteria. The patient was eligible for TCAR, and it was performed in 2024 under general anesthesia at the Houston Methodist DeBakey Heart & Vascular Center (Figure 2).

**Figure 3:**
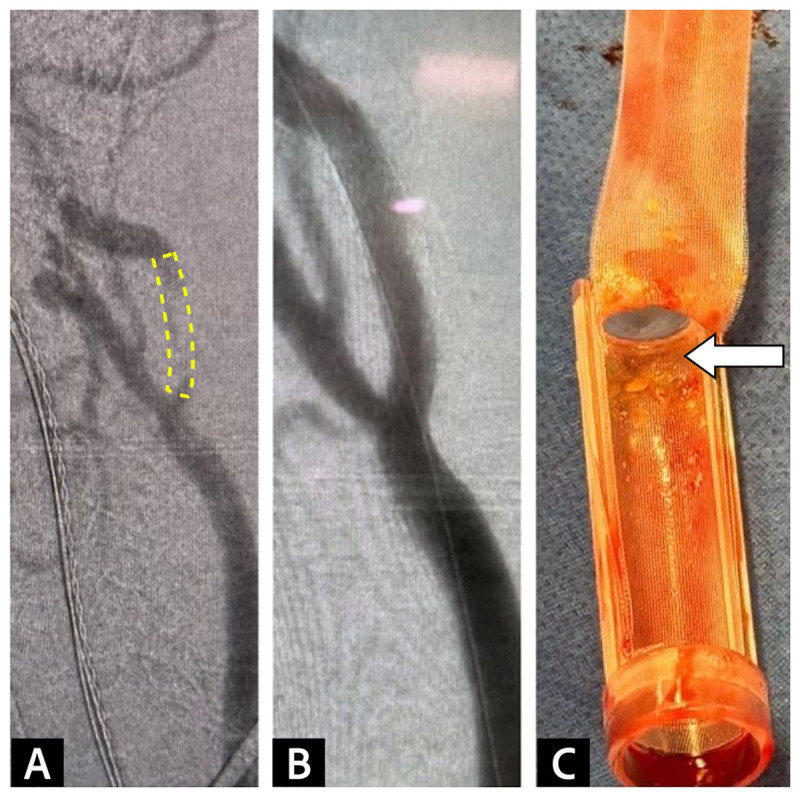
**(A)** Angiogram before stenting demonstrated stenosis in the internal carotid artery. **(B)** Post-stenting angiogram confirmed stenosis resolution. **(C)** The 200-micron filter integrated into the flow reversal system captured embolic debris before it reached the venous return sheet.

During TCAR, the surgeons placed the access sheath into the CCA and connected it with the venous sheath placed in the contralateral femoral vein. The reverse-flow transcarotid neuroprotective system was set to high speed. This redirected the blood flow from the ICA and ECA into the femoral vein. The stenotic ICA lesion was visualized by an initial angiogram, which was used to create a baseline vascular map for the surgeon (Figure 3A). After dilating the stenotic ICA lesion, a 7-mm by 40-mm ENROUTE stent was placed from the proximal ICA across the lesion, extending down to the distal CCA. Under fluoroscopic guidance, a post-dilation angioplasty was performed using a 5-mm balloon. Biphasic angiography confirmed that the stent had adequately expanded and was positioned correctly, with less than 20% residual stenosis. Meanwhile the reverse flow was continued to ensure complete removal and capture of the debris from the stenotic lesion.

TCD was used to monitor the patient during the operation. The baseline blood flow in the MCA measured 38 cm/sec and remained relatively stable despite the implementation of the reverse-flow system and clamping of the CCA. Additionally, no embolic signals were detected during the times of the wire crossing the lesion, stent deployment, or post-stent ballooning. A few HITS were observed during the arteriograms, but these were considered to be air emboli resulting from the inadequate removal of air from the syringe containing the contrast agent. Following TCD feedback, the surgical team adjusted the contrast administration technique, which reduced the frequency of the HITS. TCD revealed substantial improvement in the systolic upstroke after ICA stent placement and a post-stenting angiogram confirmed stenosis resolution (Figures 1D, 3C).

The patient experienced no perioperative complications and was discharged home the following day. Postoperative ultrasound confirmed the resolution of the stenotic lesion in the left ICA (Figure 1B).

## Discussion

Although CEA remains the most common procedure for surgically resolving carotid stenosis, TCAR offers a safe and minimally invasive alternative. As this case has demonstrated, TCAR provides three layers of protection against cerebral emboli compared to the transfemoral catheter approach. Because the access point is directly through the CCA, there is no need for manipulating intraluminal wires inside the aortic arch, which is a major source of emboli in the transfemoral approaches. Additionally, the reverse-flow method directs dislodged plaques away from the cerebral circulation. Lastly, the integration of a filter into the reverse-flow system captures the majority of the embolic debris before it reaches the venous-return sheath. In this case, the filter effectively removed debris larger than the filter pore size (Figure 3B). Furthermore, no emboli were detected during the wire crossing in the ICA lesion, stent deployment, or post-stent balloon dilation. These factors make this case a great example of optimal cerebral protection during a procedure to treat carotid artery disease.

During the procedure, there were no major drops in the MCA after reverse-flow implementation. Preserved perfusion was expected due to the robust collateral circulation from the contralateral ICA system, as revealed by the preoperative TCD examination. Some studies have suggested that an incomplete anterior circulation collateral pathway in the circle of Willis, as observed on CTA, may correlate with a significant drop in flow velocities following the initiation of flow reversal.^16^

Considering this, integrating preoperative TCD findings with MRA and CTA images enhances confidence in predicting whether the patient will tolerate the reverse flow. At Houston Methodist, there is a 50% tolerance for MCA drops during carotid procedures. Giller et al. have shown that a decline exceeding 65% is considered significant.^17^ However, there is not always a correlation between drops in MCA flow during procedures and postoperative stroke. In one study involving 11 patients undergoing TCAR, the only patient who experienced a stroke within 30 days after the procedure had a drop in MCA velocity of only 5 cm/sec.^16^ Therefore, the risk factors for stroke after TCAR need to be further elucidated.

## Limitations

There are limitations to the neuroprotective system. It does not filter particles smaller than the pore size, thus allowing sub-200-micron emboli to pass through to the venous side. Additionally, reverse flow can only be established in patients with collateral circulation robust enough to support the side of the lesion from the contralateral arterial system. Furthermore, TCD cannot differentiate between solid and gaseous emboli, although this feature would be helpful during interpretation. Nonetheless, the measurement of the maximum amplitude of the Doppler signal and its utility in differentiating between various types of embolic materials has been described in the literature.^18^ Moreover, HITS detected during contrast agent injection are most likely due to air remaining in the syringe, thereby causing air emboli. Likewise, HITS detected during the time of wire crossing through the stenotic lesion can be interpreted as solid emboli with a high degree of certainty. In this case only the left MCA was able to be monitored due to the positioning of the patient’s head during the procedure.

## Conclusion

TCD monitoring during TCAR not only measures flow velocity in the cerebral arteries but also detects emboli during wire crossing, stent deployment, and post-stent balloon dilation. The reverse blood flow in the ICA and the filter system provide neuroprotection during the procedure, reducing the risk of perioperative stroke. Lack of adequate collateral circulation may lead to a marked drop in flow dynamics once the reverse flow is initiated. However, preoperative TCD and CTA imaging are valuable tools for assessing the state of collateral circulation. When chosen appropriately for patients, TCAR provides low perioperative and long-term risks of stroke and mortality comparable to those of CEA. However, TCAR offers the advantage of a minimally invasive approach with shorter procedure and recovery times.
